# Use of reverse osmosis concentrate for mitigating greenhouse gas emissions from pig slurry

**DOI:** 10.3389/fmicb.2023.1180018

**Published:** 2023-05-17

**Authors:** Seongwon Im, Sungwon Kang, Duksoo Jang, Gyeongchul Kim, Dong-Hoon Kim

**Affiliations:** ^1^Department of Environmental Research, Korea Institute of Civil Engineering and Building Technology, Goyang-si, Gyeonggi-do, Republic of Korea; ^2^Department of Global Smart City, Sungkyunkwan University (SKKU), Suwon-si, Gyeonggi-do, Republic of Korea; ^3^Department of Civil Engineering, Inha University, Incheon, Republic of Korea

**Keywords:** greenhouse gas emissions, pig slurry, salt, reverse osmosis concentrate (ROC), biogas production

## Abstract

Due to the high global warming potential (GWP) in a short time scale (GWP100 = 28 vs. GWP20 = 86), mitigating CH_4_ emissions could have an early impact on reducing current global warming effects. The manure storage tank emits a significant amount of CH_4_, which can diminish the environmental benefit resulting from the anaerobic digestion of manure that can generate renewable energy. In the present study, we added the reverse osmosis concentrate (ROC) rich in salt to the pig slurry (PS) storage tank to reduce CH_4_ emissions. Simultaneously, pure NaCl was tested at the same concentration to compare and verify the performance of ROC addition. During 40 days of storage, 1.83 kg CH_4_/ton PS was emitted, which was reduced by 7–75% by the addition of ROC at 1–9 g Na^+^/L. This decrease was found to be more intensive than that found upon adding pure sodium, which was caused by the presence of sulfate rich in ROC, resulting in synergistic inhibition. The results of the microbial community and activity test showed that sodium directly inhibited methanogenic activity rather than acidogenic activity. In the subsequent biogas production from the stored PS, more CH_4_ was obtained by ROC addition due to the preservation of organic matter during storage. Overall, 51.2 kg CO_2_ eq./ton PS was emitted during the storage, while 8 kg CO_2_ eq./ton PS was reduced by biogas production in the case of control, resulting in a total of 43.2 kg CO_2_ eq./ton PS. This amount of greenhouse gas emissions was reduced by ROC addition at 5 g Na^+^/L by 22 and 65 kg CO_2_ eq./ton PS, considering GWP100 and GWP20 of CH_4_, respectively, where most of the reduction was achieved during the storage process. To the best of our knowledge, this was the first report using salty waste to reduce GHG emissions in a proper place, e.g., a manure storage tank.

## 1. Introduction

Global climate change and its effects are increasing, with climate-related disasters such as heat waves, droughts, floods, and wildfires piling up season after season (Nakano, 2021). Many countries have set the target of “carbon net zero” until 2040 or 2050, but some scientists warn that the time has already arrived to act “urgently now” to reduce greenhouse gas (GHG) levels in the atmosphere, not after several years (Qin et al., 2021). Methane (CH_4_) is the second-largest GHG on earth, contributing to 17.3% of global emissions (IPCC, 2013). Its main source is the agriculture industry and fugitive emissions, and it has a global warming potential (GWP100) value of 28 over 100 years, relative to carbon dioxide (CO_2_). However, CH_4_ has a much higher global warming impact in a short time scale as follows: GWP value of 86 over 20 years (IEA, 2020). This fact indicates the importance of mitigating CH_4_ emissions to have an early impact on alleviating current global warming effects (Arora and Mishra, 2021).

However, CH_4_ is a well-known renewable energy source when it is recovered from organic wastes in a biogas plant or a landfill site (Appels et al., 2008; Zhang et al., 2022). It can be biologically produced under anaerobic conditions and is further used for electrical energy generation or alternative natural gas sources. The main research area in biogas has been increasing CH_4_ production potential from various organic wastes (Wehner et al., 2021). However, it is important to point out that “reducing CH_4_ emissions could have higher significance than producing more in the life-cycle perspective, in case of e.g., livestock manure” (Im et al., 2021). In other words, by reducing 1 kg of CH_4_ emissions and producing 1 kg of more CH_4_, we can reduce GHG emissions by 28 kg CO_2_ eq. (considering GWP100 value) and 2 kg CO_2_ eq., respectively (refer to the detailed calculation procedure in the Section 2).

Pig farms emit a significant amount of CH_4_, in particular, from the storage tank (Svane and Karring, 2022). For example, pig slurry (PS) generally stays for 1–6 months before being transported to treatment facilities (Riaño and García-González, 2015; Loyon, 2018). PS can be piled up several meters, creating an anaerobic condition, and emits 1.1–4.2 kg of CH_4_/ton of PS during the storage period (Clemens et al., 2006; Misselbrook et al., 2016; Petersen et al., 2016; Im et al., 2022). In the life-cycle analysis, this amount of emissions could diminish the environmental benefit resulting from the anaerobic digestion (AD) of PS, which can generate renewable energy (Shin et al., 2019; Im et al., 2020). To reduce CH_4_ emissions, acidification with strong chemicals, mostly sulfuric acid, has often been tested and applied (Petersen et al., 2012). However, there are some safety issues in its handling, and the preparation of acids could add another carbon footprint (Im et al., 2021).

Salty waste such as reverse osmosis concentrate (ROC) from the desalination plant is rich in sodium, having a concentration of more than 25 g Na^+^/L (Woolard and Irvine, 1995; Panagopoulos, 2022). ROC is currently just discharged to the sea without treatment, possibly destroying aquatic ecosystems (Missimer and Maliva, 2018). Meanwhile, vulnerable characteristics of methanogenic consortiums are well reported, while their activity is reduced by half at 4–13 g Na^+^/L (Rinzema et al., 1988; Feijoo et al., 1995). Therefore, the addition of ROC to the PS can inhibit the indigenous methanogenic activity during the storage period, resulting in fewer CH_4_ emissions. Since ROC is a waste, it is free from adding an extra carbon footprint and easier to handle compared to strong acids. However, ROC addition to the PS can lower the subsequent AD efficiency, which needs to be checked with a systematic environmental analysis.

In the present study, we tested the ROC addition effect on reducing CH_4_ emissions during the storage of PS (30°C for 40 days), and the results were compared with those obtained by adding pure sodium (1–13 g Na^+^/L). At the same sodium concentration level, ROC addition showed higher performance in reducing CH_4_ emissions than pure sodium, and the reasons were revealed through the additional experiment. Since CH_4_ production can be suppressed by the inhibition of either bacteria or methanogens, microbial community and activity tests were performed for a better understanding of the inhibition effect. Then, the biogas potential of stored PS was obtained from the operation of continuous AD reactors. Finally, based on the amount of CH_4_ emissions during storage and biogas production in AD, the environmental assessment was made while considering both GWP100 and GWP20 values of CH_4_. To the best of our knowledge, this was the first attempt at using salty waste to reduce GHG emissions in a proper place, e.g., a manure storage tank.

## 2. Materials and methods

### 2.1. Pig slurry, seed sludge, and reverse osmosis concentrate

Pig slurry was collected from the pit at a local farm that raises 15,000 pigs in Nonsan City, Republic of Korea. It was transported in a 20-L jar packed with ice to the laboratory in 3 h and immediately used for the storage experiment. The concentrations of total solids (TS), volatile solids (VS), and chemical oxygen demand (COD) for raw PS were 76.9 ± 2.7 g/L, 51.0 ± 1.7 g/L, and 78.4 ± 2.3 g/L, respectively.

The seed sludge for the operation of continuous AD reactors was obtained from a municipal wastewater treatment plant in Incheon, Korea. Prior to its use, large particles of the seed sludge were discarded by sieving using a 10 mesh sieve, and the remaining sludge was stored in the refrigerator at 4°C. The concentrations of TS, VS, COD, and pH for the seed sludge were 27.8 ± 0.8 g/L, 25.3 ± 0.4 g/L, 37.7 ± 1.1 g/L, and 7.4 ± 0.1, respectively.

The ROC was obtained from a pilot-scale desalination plant in Jeju Island, Korea, with a capacity of producing 100 ton/day. The RO system was operated at 50% recovery. The major ions and metals contained in ROC were measured and are presented in Table 1. As a typical ROC, it was rich in Na^+^, Mg^2+^, Cl^−^, and ${\text{SO}}_{4}^{2-}$ (Xu et al., 2018).

**Table 1:** The concentrations of main cations, anions, and metals contained in reverse osmosis concentrate and pig slurry.

**Samples**	**Ion (mg/L)**						
	**Na** ^+^	**K** ^+^	**Mg** ^2+^	**Ca** ^2+^	**NO** ${}_{3}^{-}$	**Cl** ^−^	**SO** ${}_{4}^{2-}$
ROC	28,510	3,200	4,510	1,220	-	36,000	6,280
Pig slurry	510	2,310	1,840	5,930	1,130	760	510
**Samples**	**Metal (mg/L)**						
	**Al**	**Fe**	**B**	**Si**	**Cr**	**Mn**	**Ni**
ROC	1	4	31	37	-	-	-
Pig slurry	68	627	1	71	1	78	-
**Samples**	**Metal (mg/L)**						
	**Cu**	**Zn**	**As**	**Sr**	**Cd**	**Ba**	
ROC	-	-	-	19	-	-	
Pig slurry	82	250	-	7	-	5	

### 2.2. Storage experiment

A cylindrical acrylic tank with an effective volume of 1.0 L was used for storage. First, 0.65 L of PS was added to the tank, and considering the intrinsic sodium concentrations of PS (0.5 g/L) and ROC (28.5 g/L), a certain amount of pure NaCl (99.9%) or ROC was added to reach the targeting sodium concentration of 1–13 g Na^+^/L. For example, 0.234 L (= {7.0–(0.51 × 0.65)}/28.51) of ROC was added to 0.65 L of PS for adjusting the sodium concentration to 7 g Na^+^/L. Since the ROC contains not only sodium but also many other components, as shown in Table 1, different results could be obtained at the same sodium level. The rest of the volume was filled with tap water. All tanks were submerged in a water bath where the temperature was controlled at 30 ± 1°C. The gas composition of the sampled gas was analyzed every 2–5 days, and the headspace of the tanks was purged with fresh air by using a peristaltic pump (flow rate 1.0 L/min, 5 min) after gas analysis to provide actual storage condition (Shin et al., 2019). All experiments were carried out in duplicate, and the results were averaged.

### 2.3. Operation of continuous digesters

For measuring the CH_4_ potential of stored PS at different sodium concentrations (no addition, 3 g Na^+^/L, 5 g Na^+^/L, and 7 g Na^+^/L adjusted by ROC addition), four 1-L Duran glass bottles (total volume 1.2 L) were used as AD reactors. In other words, the PS stored in the former experiment for 40 days was initially moved to the refrigerator to stop further reaction and was used as feedstock in the continuous AD reactors. The prepared seed sludge was filled to reach the working volume (0.6 L), and the headspace of reactors was purged with nitrogen (99.99%) for 5 min at a flow rate of 10 L/min. When the intrinsic biogas production almost ceased, the stored PS added with ROC was supplied at a fixed hydraulic retention time of 20 days, corresponding to the organic loading rate of 1.5–1.9 g COD/L/day (e.g., 31.5 g COD/L × (20 days)^−1^ for the control). The initial VS concentration of the digestate in the four reactors was 17.5–18.2 g/L, which was lower than that of the fresh seed sludge. All digesters were operated in a shaking incubator (37 ± 1°C and 120 rpm). Biogas production and CH_4_ content were measured daily. CH_4_ production yield (MPY) was calculated with the volume of CH_4_ production (ml) and the amount of only PS addition (0.6 kg/20 days × 0.65/1.0) but not ROC and tap water.

### 2.4. Microbial activity test

#### 2.4.1. Specific acidogenic activity

The specific acidogenic activity (SAA) test was performed to investigate the sodium inhibition on the bacterial activity in PS. Glass serum bottles (total volume 270 ml) with a working volume of 100 ml were used. Glucose and the stored PS were used as the main substrate and inoculum, respectively. The biomass concentration was adjusted to 1.5 g/L (volatile suspended solid (VSS) basis), which was obtained from the control and salt-added PS (3, 5, and 7 g Na^+^/L) after 40 days of storage. The substrate was added at a substrate-to-inoculum ratio of 1 g COD/g VSS. Afterward, other nutrients and trace metals were added according to the previous study (Im et al., 2020). The bottles containing the substrate, inoculum, and nutrients were sealed using rubber stoppers secured with aluminum crimps. Their headspace was flushed following the preparation of the continuous digester operation, and the prepared bottles were placed in an incubator. Temperature and shaking speed were controlled at 37 ± 1°C and 120 rpm, respectively. Liquid samples in the bottles were taken every 4 h to determine the remaining substrate concentration during 24-h experiments. The SAA test was carried out in triplicate, and the results were averaged.

#### 2.4.2. Specific methanogenic activity

To check whether the methanogens were directly inhibited by sodium addition, specific methanogenic activity (SMA) was carried out. A certain amount of stored PS (3, 5, and 7 g Na^+^/L) was added to the bottles used in the SAA test to reach the initial microbial concentration of 5 g VS/L. A total of 2.0 g COD/L of sodium acetate or 245 ml of H_2_/CO_2_ (mixing ratio = 4:1) were added to the bottles as an electron donor (Pereira et al., 2004). The next procedure was followed by one of the SAA tests. Biogas volume and composition were analyzed until its production was stopped. The experiments were carried out in triplicate, and the results were averaged. To analyze the cumulative CH_4_ production curve, the modified Gompertz model (Equation 1) was applied to determine the CH_4_ production rate and lag period (Bianco et al., 2021).


(1) $$\begin{array}{l} \text{M}\left(t\right)={\text{M}}_{0}\times \text{exp }\left\{-\exp\left[\frac{{\text{R}}_{0}\times \text{e}}{{\text{M}}_{0}}\times \left(\lambda -\text{t}\right)+1\right]\right\}, \end{array}$$

Where M(t) = cumulative CH_4_ production at cultivation time t (ml); M_0_ = the CH_4_ production potential (ml); R_0_ = the CH_4_ production rate (ml/day); λ = the lag period (d), and e is 2.71828.

### 2.5. Analytical methods

#### 2.5.1. Liquid and solid characteristics

pH and concentrations of TS, VS, COD, and VSS were measured according to standard methods (APHA et al., 2012). The volume of biogas production was measured using a gas syringe or a gas collector and was converted to standard temperature and pressure (STP). The CH_4_ and CO_2_ contents were measured using gas chromatography (Series 580, GowMax Instrument Co.) equipped with a thermal conductivity detector (TCD) and a 1.8 m × 3.2 mm stainless-steel column packed with porapack Q (80/100 mesh SS). Nitrogen (N_2_, 99.999%) was used as the carrier gas with a flow rate of 30 ml/min, and the temperatures of the injector, column, and detector were 70, 50, and 80^°*r*^C, respectively. The concentrations of ions and metals contained in the ROC and PS were analyzed using ICP-OES (Optima 7300DV, PerkinElmer).

#### 2.5.2. Microbial community analysis

For observing the variation of bacteria and archaea consortiums, the samples for bacterial and archaeal community analyses were collected from the PS stored at 3, 5, and 7 g Na^+^/L after 40 days of storage and analyzed by the next-generation sequencing (NGS) method by a commercial sequencing facility (Macrogen, Seoul, South Korea). The next procedure was referred from the previous study by Im et al. (2020).

### 2.6. Calculations

#### 2.6.1. Inhibiting concentration

Inhibition assays for methanogenic reactions were performed with the experimental results from the storage experiment (refer to the “CH_4_ emissions” section). The CH_4_ production rate was calculated using the modified Gompertz model as described in the SMA test. The sodium concentration was 1–13 g Na^+^/L for salt-added PS and 1–9 g Na^+^/L for ROC-added PS. The inhibition effect of sodium on methanogenic activity was analyzed using a non-competitive inhibition model with CH_4_ production rate (Equation 2) (Han and Levenspiel, 1988).


(2) $$\begin{array}{l} R=R_{0}{\left(1-\frac{I}{I^{*}}\right)}^{n}, \end{array}$$

Where “*R*” is the CH_4_ production rate at inhibitor concentration of *I* (ml CH_4_/day); “*R*_0_” is the maximum CH_4_ production rate (without inhibitor) (ml CH_4_/d); “*I*” is inhibitor concentration (g Na^+^/L); “I^*^” is the lethal inhibitor concentration beyond which the reaction cannot proceed (g Na^+^/L); and “*n*” is constant (2.1249 for salt addition and 1.0747 for ROC addition).

#### 2.6.2. Greenhouse gas emissions

The amount of GHG emissions during storage was calculated using Equation 3 (based on only emitted CH_4_):


(3) $$\begin{array}{l} GHG_{storage}=\frac{M_{1}\times GWP_{CH_{4}}}{V_{1}}\times 10^{3}, \end{array}$$

Where “*GHG*_*storage*_” is the amount of GHG emissions calculated based on CH_4_ (kg CO_2_ eq./ton PS); “*M*_1_” is the cumulative CH_4_ emissions from PS (kg CH_4_); “*GWP*_*C*_*H*__4__” is a GWP value of CH_4_ (28 and 86 kg CO_2_/kg CH_4_ for 100 and 20 years, respectively); and “*V*_1_” is a volume of PS added to the storage tank (L).

The GHG reduction by biogas production was estimated using Equation 4:


(4) $$\begin{array}{l} GHG_{biogas\;production}=\frac{M_{2}\times LHV_{CH_{4}}\times CF\times \eta \times GHG_{e}}{V_{2}}\times 10^{3}, \end{array}$$

Where “*GHG*_*biogas production*_” is the amount of GHG emissions avoided by biogas production (kg CO_2_ eq./ton PS); “*M*_2_” is the amount of CH_4_ produced by the operating AD reactor (kg CH_4_); “*LHV*_*C*_*H*__4__” is the lower heating value of pure CH_4_ (50.1 MJ/kg CH_4_) (Cuéllar and Webber, 2008); “*CF*” is a conversion factor from megajoules to kilowatt hours (0.278 kWh/MJ); “η” is a conversion efficiency of electrical energy generation by biogas combustion (0.3) (Cuéllar and Webber, 2008); “*GHG*_*e*_” is a GHG emission factor for electrical energy generation from coal combustion (0.46 kg CO_2_ eq./kWh) (Moriizumi et al., 2012); and “*V*_2_” is the volume of PS added to the AD reactor (L).

## 3. Results and discussion

### 3.1. CH_4_ emissions

**Figure 1:**
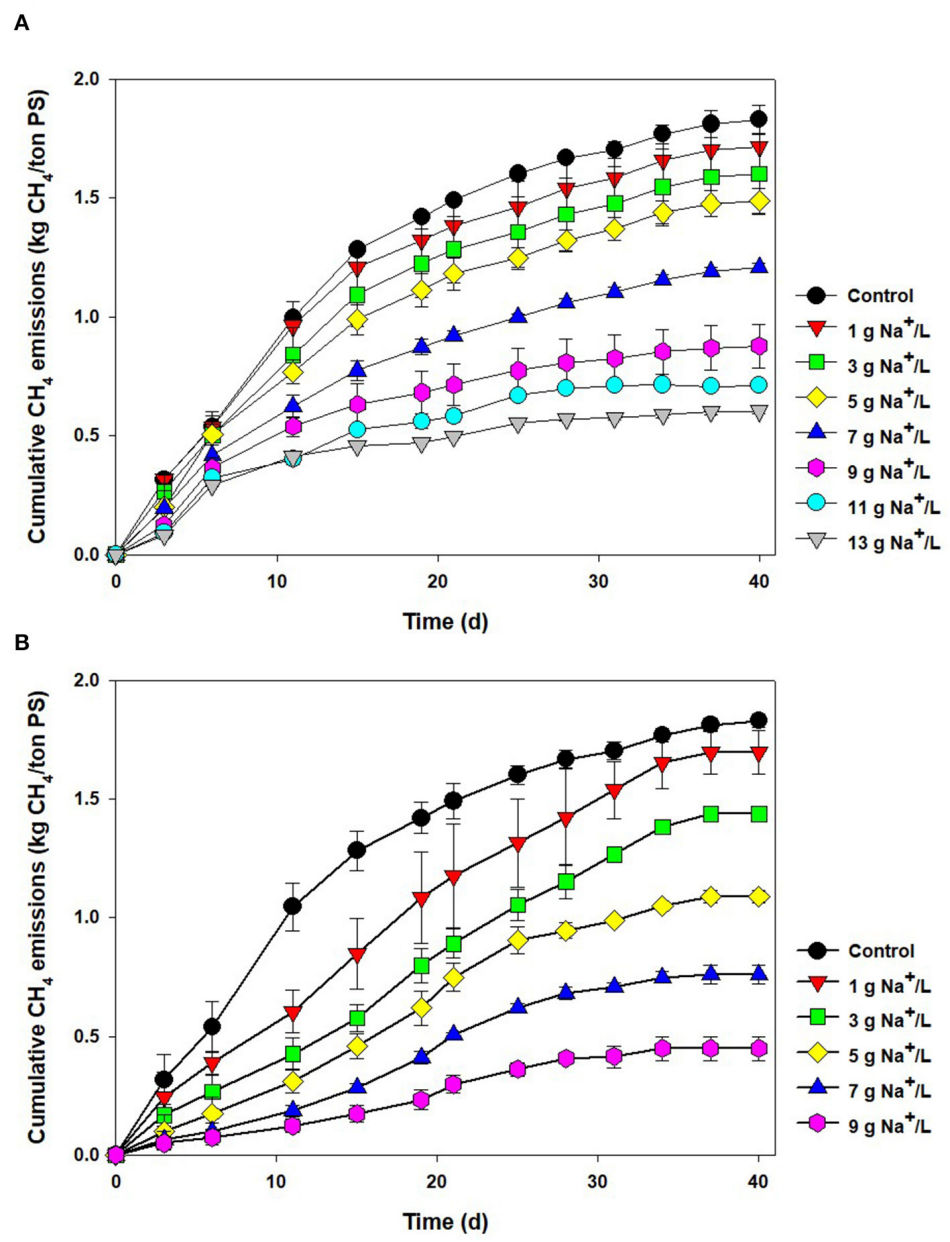
Cumulative CH_4_ emissions from the pig slurry added with **(A)** salt (1–13 g Na^+^/L) and **(B)** reverse osmosis concentrate (1–9 g Na^+^/L) during storage (40 days).

Figure 1A shows the cumulative CH_4_ emissions from the salt-added PS during 40 days of storage. During the period of storage, the amount of CH_4_ emissions from the control (raw PS) was increased, finally reaching 1.83 ± 0.06 kg CH_4_/ton PS, which was within the range of CH_4_ emissions obtained from the former works (1.5–2.5 kg CH_4_/ton PS) (Clemens et al., 2006; Chen et al., 2008; Im et al., 2022). As sodium concentration increased from 1 to 13 g Na^+^/L, the emissions gradually decreased to 1.72–0.60 kg CH_4_/ton PS, due to the well-known sodium inhibition of the methanogenic consortium (Feijoo et al., 1995). At a low concentration (like in raw PS used here), sodium is regarded as an essential element for the growth of methanogens (ex. *Methanococcus voltae*) with its role in the formation of adenosine triphosphate (ATP) or the oxidation of nicotinamide adenine dinucleotide (Dimroth and Thomer, 1989; Dybas and Konisky, 1992). However, the metabolism of microorganisms could interfere with the high sodium concentration. In particular, the increase in sodium concentration outside the microbial cell will interrupt the sodium gradient, which contributes to the driving force for ATP synthesis and energy generation (Chen et al., 2008). Previous studies used to show a proportional drop in CH_4_ productivity in AD with the increase in sodium concentration when using unacclimated biomass. Inhibition impact varied depending on the operating conditions, but generally, 50 and 100% inhibition were found at 6–10 g Na^+^/L and >15 g Na^+^/L, respectively (Rinzema et al., 1988; Lefebvre and Moletta, 2006).

**Figure 2:**
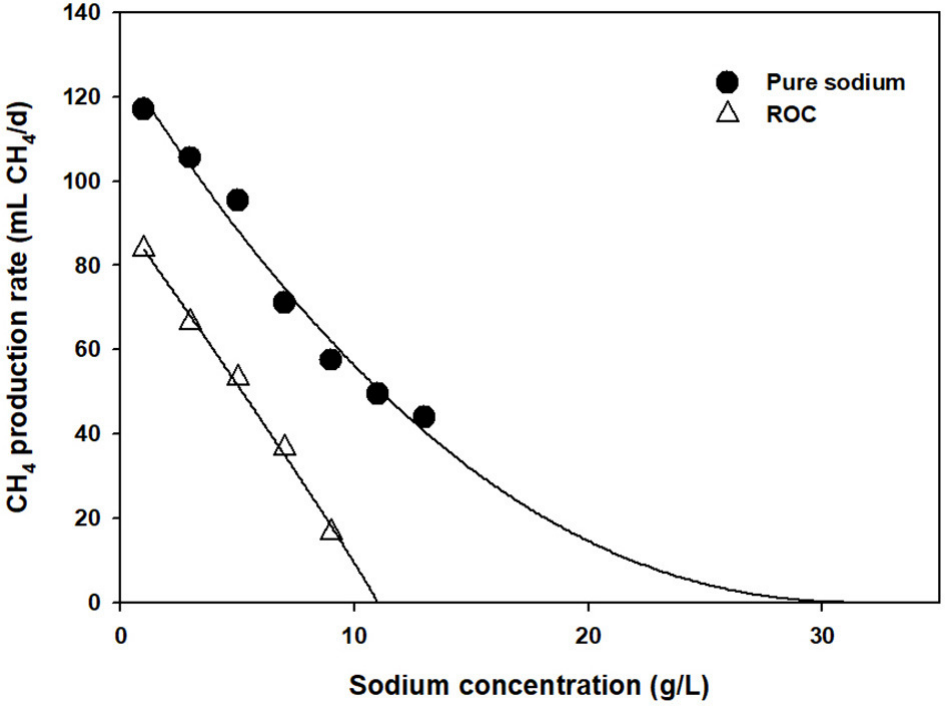
Comparison of salt and reverse osmosis concentrate inhibition assays at different sodium concentrations.

As a substitute for salt, ROC was tested, and cumulative CH_4_ emissions are shown in Figure 1B. Interestingly, it seemed that more severe inhibition was found at the same level of sodium concentration. At the sodium concentration of 1–9 g Na^+^/L, the CH_4_ emissions were reduced by 7–75%. At 7 g Na^+^/L, approximately 60% reduction in CH_4_ emissions was observed in the ROC-added bottle, while it was only 34% in the pure sodium-added one. To compare this phenomenon explicitly, CH_4_ production rates were obtained at each condition using a modified Gompertz equation and fitted by a noncompetitive inhibition model (0.99 > R^2^). As clearly shown in Figure 2, the slope of inhibition by ROC addition was steeper than that of the pure-salt added case, with different “I^*^” values of 31.08 (pure sodium) and 11.40 g Na^+^/L (ROC). The concentrations that caused inhibition by 30, 50, and 70% were 3.22, 5.42, and 7.68 g Na^+^/L for ROC addition and 4.80, 8.65, and 13.44 g Na^+^/L for pure sodium addition, respectively. The reason for the above result could be related to “synergistic inhibition” due to the existence of other ions present in ROC. This would be further discussed in the “Synergistic inhibition by reverse osmosis concentrate addition” section.

Due to ROC and tap water addition, the initial VS and COD concentrations were 33–35% lower than the raw PS (Table 2). After 40 days of storage, the VS and COD concentrations in the control were reduced by 49 and 38%, respectively, while the reduction efficiency gradually decreased as the sodium concentration increased. At 7 g Na^+^/L, the VS and COD concentrations were reduced by only 34 and 26%, respectively, which directly indicates the inhibition of organic matter degradation in the PS. Similar to the ROC addition, organic matter was also preserved by adding pure salt (Supplementary Table 1). For example, VS and COD concentrations were reduced by 40 and 30% at 7 g Na^+^/L, respectively, and the minimum values were obtained at 13 g Na^+^/L (VS 36% and COD 21%, respectively). Preservation of organic matter was previously reported in testing the acidification method. During 30–40 days of storage, VS and COD concentrations were reduced by 30–40% under common storage conditions, but they were decreased to only 3–30% by H_2_SO_4_ addition depending on the acidification strength (Shin et al., 2019; Im et al., 2021).

**Table 2:** Characteristics of diluted raw and stored pig slurry added with reverse osmosis concentrate at different sodium concentrations (1–9 g Na^+^/L).

**Parameter**	**Initial pig slurry**	**After storage (g Na** ^**+**^ **/L)**			
		**Control**	**1**	**3**	**5**	**7**	**9**
TS (g/L)	50.4 ± 1.2	27.8 ± 1.4	30.5 ± 1.9	30.5 ± 1.5	34.0 ± 1.0	37.7 ± 0.4	40.1 ± 0.4
VS (g/L)	33.7 ± 2.1	17.2 ± 0.7	19.2 ± 1.4	19.1 ± 0.9	20.0 ± 0.8	22.2 ± 0.3	22.3 ± 0.3
COD (g/L)	51.0 ± 2.3	31.5 ± 2.3	33.3 ± 1.7	34.0 ± 2.2	35.3 ± 3.4	37.8 ± 1.8	39.9 ± 2.3

The suppression of the loss of organic matter can be attained through inhibiting methanogenesis, end-step of anaerobic degradation, and aerobic degradation. The proportion of each pathway could be estimated using the amount of COD loss from the PS and CH_4_ emissions. For 40 days of storage, 19.5 g of COD was removed in the control, which was decreased to 11.1–17.7 g of COD at 1–9 g Na^+^/L [e.g., (51.0–31.5) g COD/L × 1 L for the control]. In cases of CH_4_ emissions, 1.19 g and 0.29–1.10 g of CH_4_ were emitted in the control and the ROC-added PS, respectively, corresponding to 4.76 g and 1.17–4.41 g of COD, respectively (e.g., 1.83 g CH_4_/kg × 0.65 kg × 22.4 L CH_4_/16 g CH_4_ × 100/35 g COD/L CH_4_ for the control). By subtracting COD loss and the mass of COD value converted into CH_4_ emissions, the amount of COD loss by aerobic degradation was 14.74 g in the control, and the value was gradually reduced to 9.93–13.29 g as the sodium concentration increased. These results implied that aerobic degradation was also inhibited by ROC addition, but the impact of sodium inhibition on aerobic degradation tended to be less significant than methanogenesis.

### 3.2. Synergistic inhibition by reverse osmosis concentrate addition

Ions and metal contents of ROC and raw PS were analyzed to find out the reasons for the synergistic effect on reducing CH_4_ emissions (Table 1). Various ions and metals that are known to affect the activity of methanogens were found in the ROC. For example, K^+^, Mg^2+^, and Ca^2+^ were observed at high concentrations, which are known to have antagonistic effects on reducing ammonia and sodium inhibition (Braun et al., 1981; Hendriksen and Ahring, 1991). As a synergistic inhibitor on CH_4_ emissions, ${\text{SO}}_{4}^{2-}$ was found and contained a high concentration in the ROC (Petersen et al., 2012). The effect of K^+^, Ca^2+^, and heavy metals on CH_4_ emissions was expected to be negligible due to their similar or low concentration in ROC compared to that of PS (Table 1). Therefore, we added Mg^2+^ and ${\text{SO}}_{4}^{2-}$ to the pure salt-added PS to confirm the synergism.

**Figure 3:**
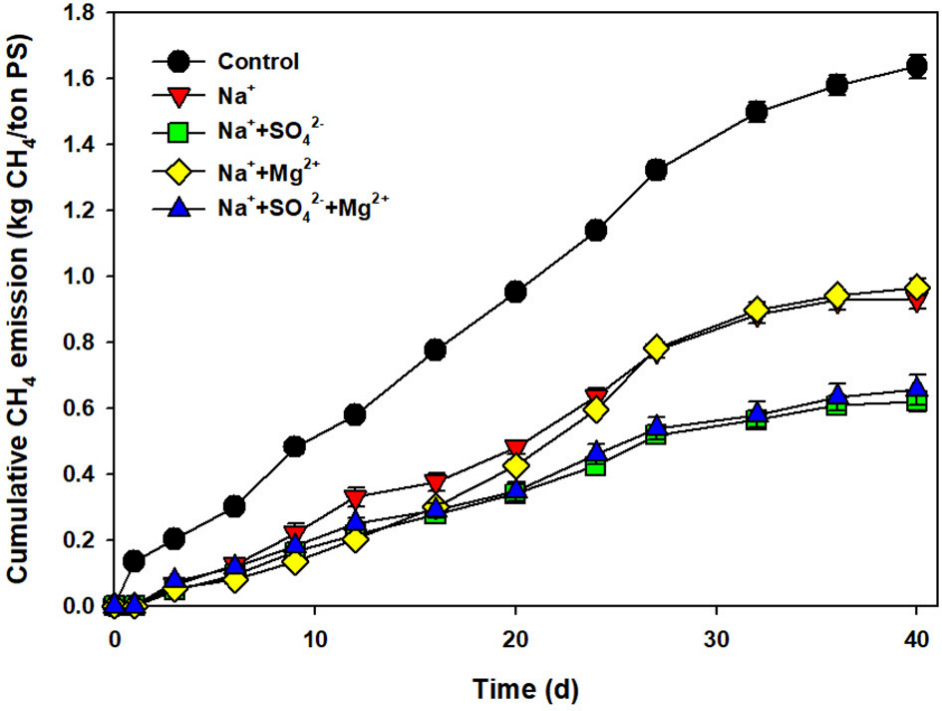
Cumulative CH_4_ emissions from the control and pig slurry added with sodium (7 g Na^+^/L), sodium + sulfate (7 g Na^+^/L + 1.5 g ${\text{SO}}_{4}^{2-}$/L), sodium + magnesium (7 g Na^+^/L + 2.8 g Mg^2+^/L), and sodium + sulfate + magnesium (7 g Na^+^/L + 1.5 g ${\text{SO}}_{4}^{2-}$/L + 2.8 g Mg^2+^/L) during storage (40 days).

As shown in Figure 3, approximately a 40% reduction in CH_4_ emissions was attained at 7 g Na^+^/L, which had a similar efficiency compared to the salt addition. A higher reduction in CH_4_ emissions of 62% was observed only in the ${\text{SO}}_{4}^{2-}$ added PS, but not by Mg^2+^. Moreover, Mg^2+^ did not play any role, with no synergism/antagonism to the sodium inhibition at the added concentration. The synergistic effect on the reduction of CH_4_ emissions by ROC addition was speculated to be due to ${\text{SO}}_{4}^{2-}$ addition. In the former study testing the effect of acidification on CH_4_ emissions, the pH drop caused by adding sulfuric acid was found to be the main mechanism to reduce CH_4_ emissions. However, it was also mentioned that the “${\text{SO}}_{4}^{2-}$ addition” can have a certain inhibitory impact on the methanogenic reaction, which was confirmed by replacing sulfuric acid with MgSO_4_ solution (Eriksen et al., 2012; Petersen et al., 2012). The effect of salt addition on CH_4_ emissions and organic matter degradation can be attributed to the change in the microbial community and microbial activity, which will be discussed in the following sections.

### 3.3. Microbial analysis

**Figure 4:**
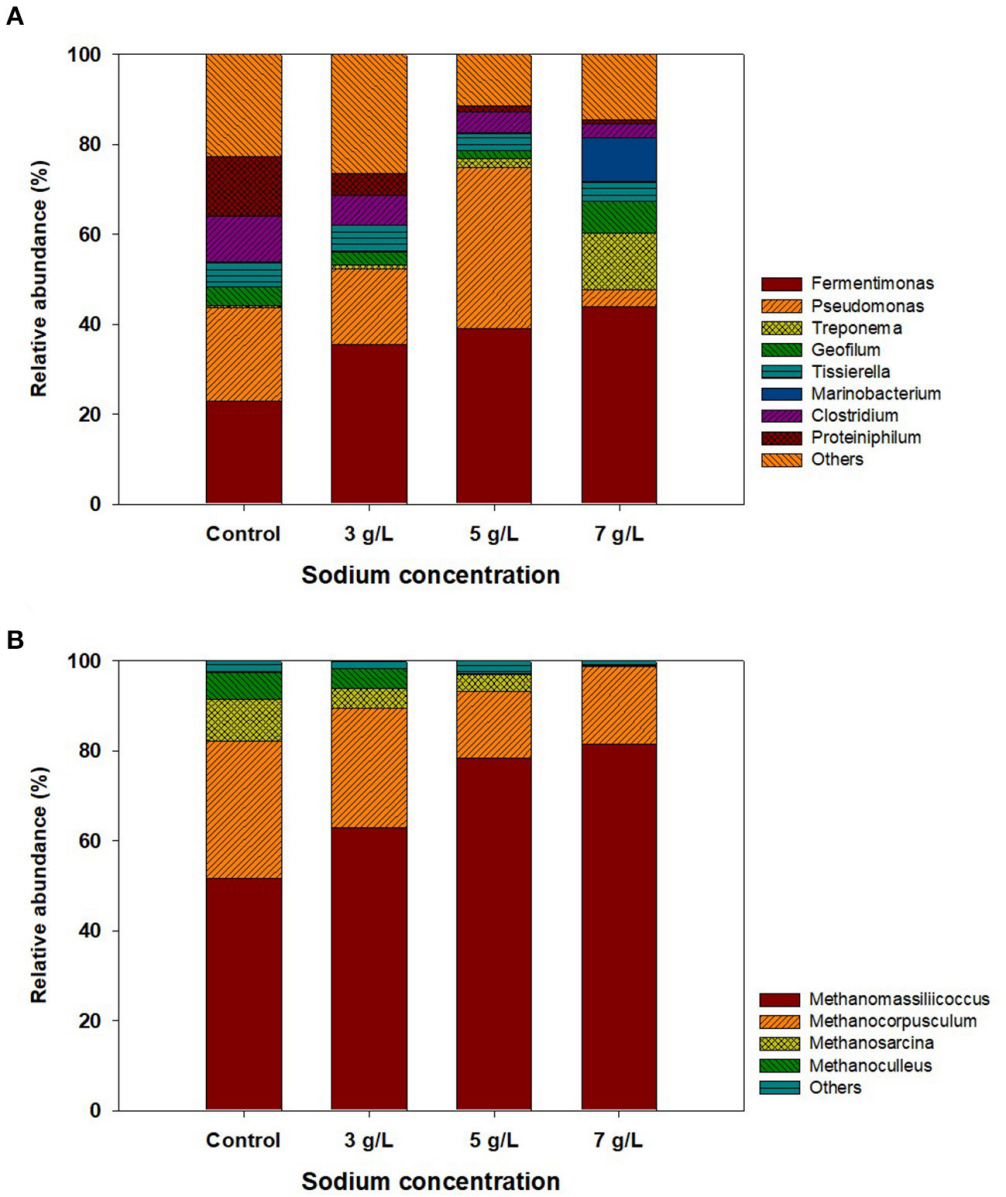
The result of next-generation sequencing analysis of stored pig slurry loaded with different sodium concentrations by adding reverse osmosis concentrate (control, 3, 5, and 7 g Na^+^/L): **(A)** bacterial and **(B)** archaeal communities.

Among five different storage conditions, the ROC-added PS at 3, 5, and 7 g Na^+^/L were collected with the control at the end of storage and analyzed by NGS to investigate the bacterial and archaeal community structure. In the bacterial community, 7, 182–17, and 274 OTUs (≥97% sequence similarity cutoff) were obtained from each sample. As shown in Figure 4A, the most abundant bacteria at the genus level in the control were *Fermentimonas* (23%), followed by *Pseudomonas* (21%), *Proteiniphilum* (13.2%), *Clostridium* (12%), and so on, which were reported to dominate in PS and/or the AD sludge fed with livestock manure (Haakensen et al., 2008; Kumari et al., 2015; Hahnke et al., 2016). Within the *Fermentimonas* group, *Fermentimonas caenicola* was found to be the most dominant species under all storage conditions, which might be related to its salt tolerance (Supplementary Table 2). The optimal sodium concentration for this strain was reported to be <1 g Na^+^/L, but its activity was observed even at approximately 10 g Na^+^/L (Hahnke et al., 2016). However, the abundance of *Pseudomonas* increased to 36% at 5 g Na^+^/L, while it decreased to 4% at 7 g Na^+^/L, probably due to exceeding the maximum sodium concentration for its growth (Xiao et al., 2009). At 7 g Na^+^/L, the abundance of *Geofilum* and *Marinobacterium* was increased to 7 and 10% and are known to grow at a wide sodium concentration range of 2–20 g Na^+^/L (González et al., 1997; Mu et al., 2017). Other species detected in the stored PS were *Treponema zuelzerae, Tissierella praeacuta, Clostridium saudiense*, and *Proteiniphilum acetatigenes*, whose abundance was slightly changed. These species are well-known acidogenic bacteria that can decompose various organic matter into small amounts of soluble organics.

The change in the archaeal community in the control and stored PS is shown in Figure 4B. In total, 66, 198-94, and 769 OTUs were obtained from each sample. The archaeal sequences were assigned by selecting four representative OTUs (≥97% similarity), which belonged to *Methanomassiliicoccus, Methanocorpusculum, Methanosarcina*, and *Methanoculleus*. These genera were often found in PS and digested sludge and were known to utilize mainly H_2_/CO_2_ as substrates (Asakawa and Nagaoka, 2003; Rea et al., 2007; Shin et al., 2019). *Methanomassiliicoccus luminyensis* was found to be a dominant member in the control, accounting for 52% of the total sequences (Supplementary Table 2). Its dominance gradually increased as the sodium concentration increased, while other methanogens (*Methanocorpusculum aggregans, Methanosarcina soligelidi*, and *Methanoculleus sediminis*) had the opposite trend in the abundance of each strain. According to previous studies, these three methanogens were commonly found in animal feces and sewage sludge and could grow at low sodium concentrations (0–4 g/L) (Xun et al., 1989; Wagner et al., 2013; Chen et al., 2015). However, it was reported that the activity and growth of *Methanomassiliicoccus luminyensis*, found as the dominant species in this study, were not inhibited at >6 g Na^+^/L, leading to an increase in its abundance. These results indicated that ROC or salt addition to PS could induce changes in the archaeal community, probably due to the increase in the abundance of viable methanogen at high sodium concentration.

The results suggested that the sodium addition somehow played a role in shifting the microbial dominance to the salt-tolerant one, indicating that the indigenous consortium changed their population to adapt to the inhibiting condition. However, there is no information on the inhibition impact for each specific microbial function. Thus, the question of whether the CH_4_ emission drop was caused by the acidogenic reaction or directly by the methanogenic reaction needs to be explored.

### 3.4. Specific acidogenic and methanogenic activity

Specific acidogenic activity and SMA tests with microorganisms obtained from the control and the ROC-added PS at 3, 5, and 7 g Na^+^/L were conducted to evaluate the effect of sodium on the specific microbial activity, and their results are summarized in Table 3. The glucose started to degrade after 13 h and was almost eliminated by 24 h (Supplementary Figure S1). The drop curve of glucose showed the same but inverse trend of producing acids. The main acids found here were acetate, butyrate, and propionate (data not shown). The maximum SAA value of 3.26 ± 0.14 g COD/g VSS/day was attained in the control, which was within the range of acidogenic activity (1–5 g COD/g VSS/day) of digester sludge treating livestock manure (Regueiro et al., 2012). By adding sodium, the SAA was reduced by 13–16% compared to the control but did not show a difference at the different sodium concentrations. These results were consistent with those of Lefebvre and Moletta (2006), who observed that the SAA value of digested sludge was decreased by only 10% at 10 g Na^+^/L. In addition, this might link to the bacterial community changes that did not show a significant trend or difference depending on the sodium concentration.

**Table 3:** Specific functional activity of microorganisms obtained from the stored pig slurry loaded with different sodium concentrations by adding reverse osmosis concentrate: no addition (control), 3, 5, and 7 g Na^+^/L (in g COD/g VSS/day).

	**SAA^a^**	**SMA** ^**b**^	
		**Acetate**	**H**_2_ **/CO**_2_
Control	3.26 ± 0.14	0.114 ± 0.010	0.064 ± 0.003
3 g Na^+^/L	2.82 ± 0.27	0.089 ± 0.007	0.058 ± 0.002
5 g Na^+^/L	2.73 ± 0.08	0.078 ± 0.005	0.053 ± 0.002
7 g Na^+^/L	2.82 ± 0.32	0.007 ± 0.003	0.014 ± 0.001

^a^SAA, Specific acidogenic activity; ^b^SMA, Specific methanogenic activity.

In both the acetate- and H_2_/CO_2_-fed SMA tests, CH_4_ production began at the beginning of the experiment in the control and PS stored at 3 and 5 g Na^+^/L and ended within 15 days, while a limited amount of CH_4_ was produced at 7 g Na^+^/L during the same experiment period with a longer lag period (Supplementary Figure S2). The maximum SMA values were acquired from the control, and the values were 0.114 ± 0.010 and 0.064 ± 0.003 g COD/g VSS/day for the acetate- and H_2_/CO_2_-fed tests, respectively (Table 3). With the increase in sodium concentration, the methanogenic activity gradually decreased by 9–94%. Similarly, Feijoo et al. (1995) showed that the SMA value of digested sludge was reduced by 20, 50, and 95% at 5, 7, and 9 g Na^+^/L, respectively. In previous studies using methanogens acclimated to high sodium concentrations over a long period, the value was slowly reduced due to the increased tolerance to sodium (Lefebvre and Moletta, 2006; Jeison et al., 2008). At the same sodium concentration, acetoclastic methanogens were likely to be more sensitive to sodium than hydrogenotrophic methanogens (Feijoo et al., 1995). For example, at 5 g Na^+^/L, the SMA value obtained from the acetate-fed test was reduced by 32%, while the reduction reached only 17% in the H_2_/CO_2_-fed test. From the results of SAA and SMA, the main reason for reduced CH_4_ emissions during storage might be the inhibition of methanogenic activity rather than bacterial activity, which was consistent with the result of organic matter degradation.

### 3.5. Biogas production potential

**Figure 5:**
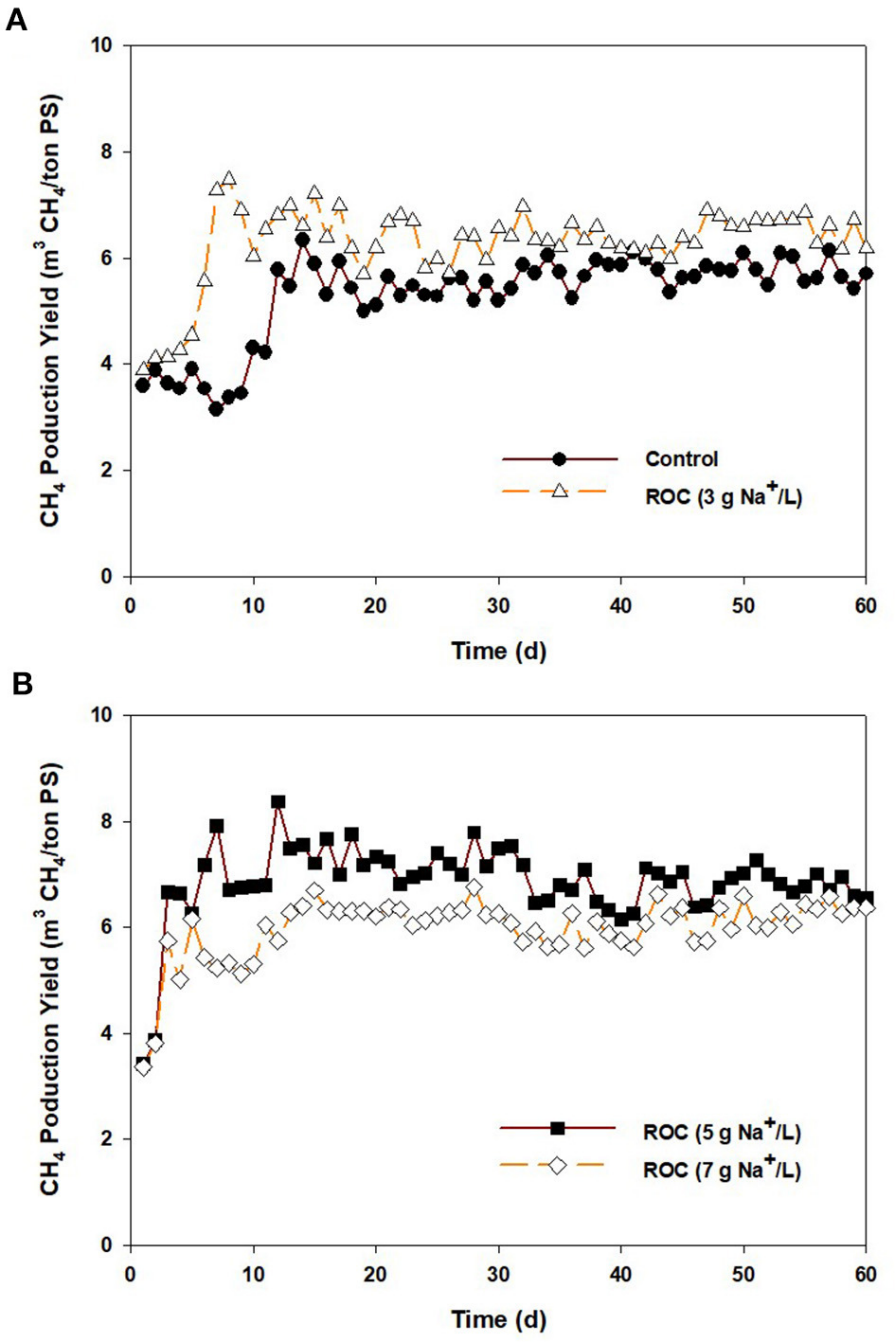
Daily CH_4_ production from anaerobic digesters fed with the stored pig slurry loaded with different sodium concentrations by adding reverse osmosis concentrate: **(A)** control and 3 g Na^+^/L, **(B)** 5 and 7 g Na^+^/L.

Figure 5 shows CH_4_ production from the control and ROC-added PS at 3, 5, and 7 g Na^+^/L. The CH_4_ content in the biogas ranged from 60 to 64% in all reactors (data not shown). The average MPY for the control was 5.7 m^3^/ton PS, and it was increased to 6.1–6.8 m^3^/ton PS at 3, 5, and 7 g Na^+^/L. In terms of CH_4_ yield (COD input basis), a similar value of 0.12 m^3^ CH_4_/kg COD_added_ (5.7–6.8 m^3^/ton PS × 0.65/1.0 × (34.5–35.3 kg COD/ton PS)^−1^) was attained from the control and 3–5 g Na^+^/L, while that was decreased to approximately 0.10 m^3^ CH_4_/kg COD_added_ at 7 g Na^+^/L. At the beginning of the operation, the CH_4_ yield of stored PS at 7 g Na^+^/L was maintained at 0.110–0.120 m^3^ CH_4_/kg COD_added_ until 30 days. Thereafter, it slightly decreased and reached 0.095–0.105 m^3^ CH_4_/kg COD_added_. Although there was a slight inhibition on CH_4_ yield at 7 g Na^+^/L, a higher MPY was obtained from the ROC-added PS than from the control, which seemed to be contrary to the reduction in CH_4_ emissions by ROC addition. Although the preservation of organics might have contributed to the enhancement of MPY, it was inadequate to explain this. Considering the CH_4_ yield for the control and the increase in COD [3.8 kg COD/ton PS = 35.3 (5 g Na^+^/L)−31.5 (control), Table 2], 0.46 m^3^ of CH_4_ can be additionally produced from 1 ton PS at 5 g Na^+^/L, which cannot cover the enhanced amount of MPY {0.66 m^3^/ton PS = [6.78 (5 g Na^+^/L)−5.76 (control)] × 0.65/1.0}. Furthermore, the increase in CH_4_ yield might not be explained by the preservation of organic matter. The expected reason for this might be the adaptation of microorganisms due to a gradual increase in sodium concentration by continuous feeding (Chen et al., 2008). According to Rinzema et al. (1988) and Lefebvre and Moletta (2006), when sufficient time is provided to adapt to high sodium concentrations, the AD process could be maintained even at >15 g Na^+^/L. In this study, ROC-added PS was slightly provided to the digester filled with non-saline digestate, and the sodium concentration was relatively low compared to the previous studies, resulting in the enhancement of MPY. However, the increase in ${\text{SO}}_{4}^{2-}$ concentrations by ROC addition might lead to the inhibition of CH_4_ production in the AD process, as shown in the “Synergistic inhibition by reverse osmosis concentrate addition” section. According to previous studies, the impact of ${\text{SO}}_{4}^{2-}$ inhibition on AD varied depending on the COD/${\text{SO}}_{4}^{2-}$ ratio of a substrate. When the ratio was higher than 5, no inhibition effect on CH_4_ yield was observed (Lu et al., 2016). However, the inhibition tended to be more intensive as the ratio lowered, and methanogenesis was significantly suppressed at a ratio of <2.0 (Om et al., 2022). The COD/${\text{SO}}_{4}^{2-}$ ratio for the stored PS samples used in the continuous AD test ranged from 61.8 to 22.5, and the minimum value was attained from the ROC-added PS at 7 g Na^+^/L, expectably. This result implied that the ${\text{SO}}_{4}^{2-}$ inhibition to the performance of continuous AD reactors might be negligible due to the high COD/${\text{SO}}_{4}^{2-}$ ratio, and the slight drop in CH_4_ yield at 7 g Na^+^ might be associated with sodium toxicity to methanogens, as shown in activity test.

### 3.6. Environmental assessment

To observe the environmental effect of ROC addition, the amount of GHG emissions/reduction during storage, the subsequent biogas (CH_4_) production, and two types of GWP were considered (Table 4). The amount of GHG reduction derived from CH_4_ production in AD can be calculated through power generation. From the control, 8.0 kg of CO_2_ eq. could be reduced *via* CH_4_ production, and slightly higher values were achieved at 3, 5, and 7 g Na^+^/L. These figures were the maximum values for reducing GHG emissions without taking into account energy (i.e., electricity) consumption for the operating plant. Assuming the mesophilic AD plant utilizes the combined heat and power unit (CHP) and common physical agitation system, approximately 20–30% of the total produced electricity might be consumed for operating them, leading to a decrease in the amount of GHG reduction through biogas production (Naegele et al., 2012). However, it is important to mention that even these maximum values were inadequate to meet “carbon net zero” because of the CH_4_ emissions during storage. Using the GWP100 value of 28, total GHG emissions from the management of PS ranged from 12.8 to 43.2 kg CO_2_ eq./ton PS. The expected amounts of GHG reduction by ROC addition were 12.0, 22.2, and 30.4 kg CO_2_ eq./ton PS at 3, 5, and 7 g Na^+^/L, respectively. In contrast, the amount of GHG emissions from PS significantly increased to 56.8–149.4 kg CO_2_ eq./ton, considering the GWP20 value of 86. In this case, the storage method, such as ROC addition, has a much higher impact on mitigating GHG emissions. The amount of GHG emissions approximately increased by 3.1 times, reaching 65.3–157.4 kg CO_2_ eq./ton PS.

**Table 4:** Total reduction in greenhouse gas emissions during storage and subsequent biogas production from the control and reverse osmosis concentrate-added pig slurry (at 3, 5, and 7 g Na^+^/L) (+: GHG emission, -: GHG reduction).

			**CH**_**4**_ **GWP** = **28**				**CH**_**4**_ **GWP** = **86**			
			**Cont**.	**3**	**5**	**7**	**Cont**.	**3**	**5**	**7**
GHG emission during storage		(kg CO_2_ eq./ton PS)	(+) 51.2	(+) 40.2	(+) 30.5	(+) 21.3	(+) 157.4	(+) 123.6	(+) 93.7	(+) 65.3
CH_4_ production	Yield	(m^3^ CH_4_ /ton PS)	5.8	6.5	6.8	6.1	5.8	6.5	6.8	6.1
	Electrical energy	(kWh/ton PS)	17.2	19.3	20.2	18.2	17.2	19.3	20.2	18.2
	GHG reduction	(kg CO_2_ eq./ton PS)	(-) 8.0	(-) 9.0	(-)9.4	(-) 8.5	(-) 8.0	(-) 9.0	(-) 9.4	(-) 8.5
Total		(kg CO_2_ eq./ton PS)	(+) 43.2	(+) 31.2	(+) 21.1	(+) 12.8	(+) 149.4	(+) 114.6	(+) 84.2	(+) 56.8

Based on this study, we concluded that sodium concentration adjustment at 5 g Na^+^/L by adding ROC might be optimal PS storage conditions concerning both the reduction of CH_4_ emissions and the enhancement of CH_4_ production. During storage, the amount of CH_4_ emissions was reduced by 40%, and MPY was increased by 18% at 5 g Na^+^/L without any inhibition of the AD process. At higher sodium concentrations, we might expect more drops of CH_4_ emissions during storage, but the volume of diluted PS will also increase. For example, approximately 380 L and 490 L of ROC are required to adjust the sodium concentration of 1 ton of PS to 7.0 and 9.0 g Na^+^/L, respectively, which is 1.4 and 1.8 times higher than in the case of 5 g Na^+^/L. Furthermore, at a sodium concentration of >5 g Na^+^/L, a drop in CH_4_ yield from the AD was observed, and saline digestate might lead to a big issue when it was finally treated by composting and applied to the field. However, no inhibition effect was observed on plant yield (leaf lettuce, potato, etc.) when the compost with a sodium concentration of 3.5–8.0 g Na^+^/L was used to grow crops (Yang et al., 2021). Therefore, we acknowledge that the use of 5 g Na^+^/L in manufacturing compost meets the national standard (Bernal et al., 2009).

To determine the practical applicability of the ROC addition, there should also be an economic assessment. However, there can be a limitation in addressing the economic aspect from the lab-scale and short-term experimental results. Moreover, the question of whether the ROC addition can have economic advantages compared to the sulfuric acid addition remains unexplored. Based on previous study results (Adeniran et al., 2017), sulfuric acid is the cheapest strong acid, costing only US$0.3–0.6/ton PS. The use of other organic acids, such as lactic acid and citric acid, will cost ten times more than using sulfuric acid (Nica and Woinaroschy, 2010). In manufacturing ROC, transportation is the only major cost and it depends on the location. Besides the hazard issue in handling, we suggest that sulfuric acid addition will cause an increase in H_2_S concentration in the subsequent biogas generation, which might increase the cost of desulfurization (Im et al., 2021). In addition, there is a chance of shortening the life length of installed equipment in the storage tank and biogas plant due to corrosion. Therefore, a long-term, full-scale experiment, including a storage tank and biogas plant, has been proposed to be operated to get the economic assessment results.

As mentioned, the ROC addition to the PS storage tank cannot be applied to the whole region. When the desalination plant is far from the pig farm, the transportation cost would impede the environmental benefit. However, in a specific region like Jeju Island in Korea, where the desalination industry is growing and the consumption of meat is increasing rapidly, the use of ROC for mitigating carbon emissions from liquid organic waste like livestock manure deserves consideration.

## 4. Conclusion

By adding ROC equivalent to 1–9 g Na^+^/L to PS, the CH_4_ emissions during the storage of PS were reduced by 7–75% compared to the control. The additional experiment proved that the presence of sulfate in ROC synergistically triggered the inhibition. Microbial community, SAA, and SMA results showed that sodium directly inhibited methanogenic activity rather than acidogenic bacterial activity. Considering the drop in CH_4_ emissions and the subsequent increased biogas production, it was concluded that ROC addition at 5 g Na^+^/L was optimal, reducing GHG emissions by 22 and 65 kg CO_2_ eq./ton PS, considering GWP100 and GWP20, respectively.

## Data availability statement

The datasets presented in this study can be found in online repositories. The names of the repository/repositories and accession number(s) can be found in the article/Supplementary material.

## Author contributions

SI designed the study, performed the experiments, processed the experimental data, and wrote the original draft. SK, DJ, and GK investigated the information regarding inhibition assays and analyzed the characteristics of ROC. D-HK contributed to the conceptualization and revision of the original draft. All authors contributed to the article and approved the submitted version.
