# Supplementary material for: Use of reverse osmosis concentrate for mitigating greenhouse gas emissions from pig slurry
Source: Front Microbiol. 2023 May 17;14:1180018. doi: 10.3389/fmicb.2023.1180018 (PMC10229891; doi:10.3389/fmicb.2023.1180018)
Supplement: Supplementary file 1 [file Data_Sheet_1.DOCX]

Supplementary Material

Use of Reverse Osmosis Concentrate for Mitigating Greenhouse Gas emissions from pig slurry

Seongwon Im, Sungwon Kang, Duksoo Jang, Gyeongchul Kim, Dong-Hoon Kim*

*** Correspondence:** Dong-Hoon Kim: dhkim77@inha.ac.kr

# Supplementary Figures

**Supplementary Figure 1.** Glucose concentration changes during specific acidogenic activity test (no addition (control), 3, 5, and 7 g Na^+^/L).


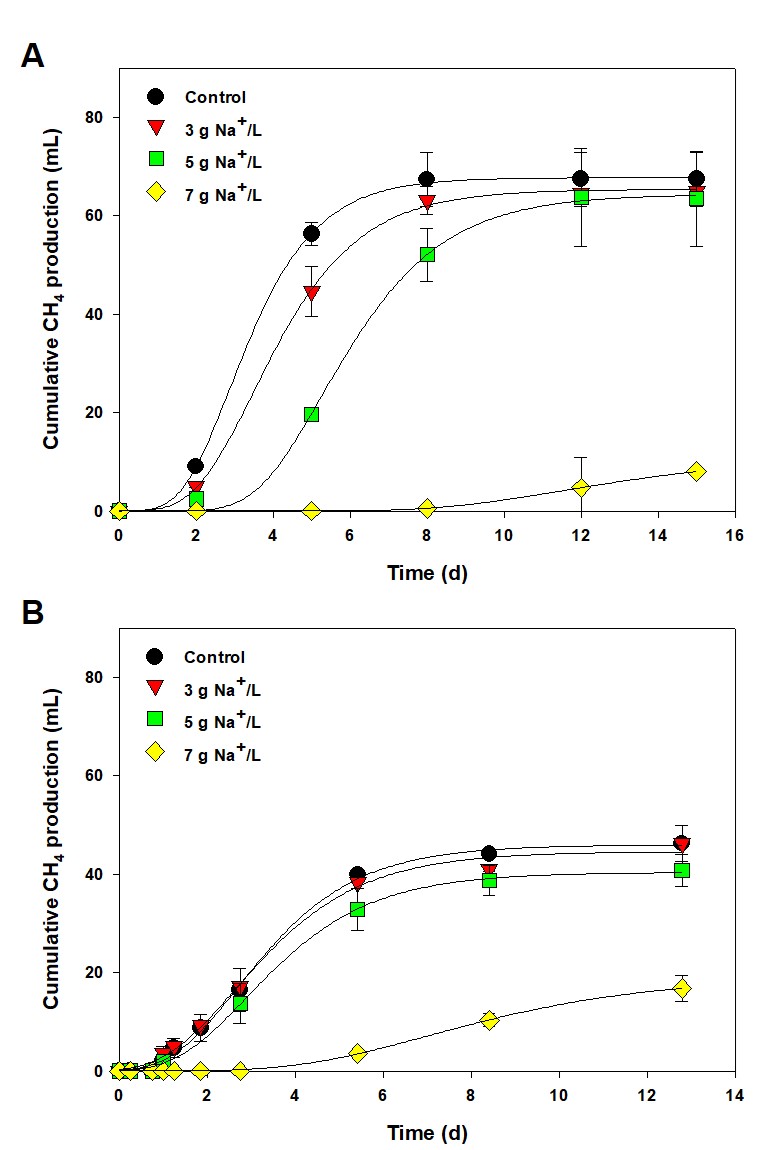


**Supplementary Figure 2.** Cumulative CH4 production during specific methanogenic activity test using (a) acetate, and (b) gas mixture of H_2_/CO_2_.

# Supplementary Tables

**Supplementary Table 1.** Characteristics of diluted raw and stored pig slurry added with salt at different sodium concentrations (1-13 g Na+/L).

| Parameter | Initial  pig slurry | After storage | | | | | | | |
| --- | --- | --- | --- | --- | --- | --- | --- | --- | --- |
|  |  | Control | 1 g Na^+^/L | 3 g Na^+^/L | 5 g Na^+^/L | 7 g Na^+^/L | 9 g Na^+^/L | 11 g Na^+^/L | 13 g Na^+^/L |
| TS  (g/L) | 50.0  ±2.7 | 27.1  ±1.0 | 28.6  ±1.4 | 29.9  ±1.1 | 31.8  ±1.0 | 34.4  ±0.3 | 36.3  ±0.4 | 37.1  ±2.6 | 39.1  ±1.2 |
| VS  (g/L) | 33.2  ±1.7 | 16.8  ±0.9 | 17.8  ±1.0 | 18.1  ±0.7 | 18.8  ±0.6 | 20.0  ±0.2 | 20.7  ±0.5 | 20.5  ±1.4 | 21.1  ±2.6 |
| COD  (g/L) | 50.9  ±2.3 | 30.5  ±2.2 | 31.8  ±2.9 | 33.3  ±1.4 | 33.6  ±2.9 | 36.0  ±3.1 | 38.0  ±4.1 | 39.0  ±3.7 | 40.2  ±5.5 |

**Supplementary Table 2.** Bacterial and archaeal communities in species level identification of the dominant sequences.

| Microorganism | | Accession number | Similarity  (%) | Control  (%) | Salt concentration  (g Na^+^/L) | | |
| --- | --- | --- | --- | --- | --- | --- | --- |
|  |  |  |  |  | 3  (%) | 5  (%) | 7  (%) |
| Bacteria | *Fermentimonas caenicola* | NR_148809.1 | 99 | 22.9 | 35.6 | 39.2 | 43.9 |
|  | *Pseudomonas caeni* | NR_116388.1 | 99 | 20.8 | 16.7 | 35.6 | 3.8 |
|  | *Treponema zuelzerae* | NR_104797.1 | 97 | 0.5 | 0.9 | 2.1 | 12.6 |
|  | *Geofilum rhodophaeum* | NR_158091.1 | 99 | 4.2 | 2.9 | 1.6 | 7.0 |
|  | *Tissierella praeacuta* | NR_044860.1 | 97 | 5.4 | 6.0 | 3.7 | 4.6 |
|  | *Marinobacterium georgiense* | NR_114163.1 | 98 | <0.1 | <0.1 | 0.3 | 9.7 |
|  | *Clostridium saudiense* | NR_144696.1 | 97 | 10.1 | 6.5 | 4.7 | 3.0 |
|  | *Proteiniphilum acetatigenes* | NR_043154.1 | 97 | 13.2 | 4.9 | 1.2 | 0.9 |
| Archaea | *Methanomassiliicoccus luminyensis* | NR_118098.1 | 98 | 51.6 | 62.9 | 78.3 | 81.5 |
|  | *Methanocorpusculum aggregans* | NR_117749.1 | 99 | 30.4 | 26.5 | 14.9 | 17.2 |
|  | *Methanosarcina soligelidi* | NR_109423.1 | 100 | 9.4 | 4.3 | 3.6 | 0.2 |
|  | *Methanoculleus sediminis* | NR_136474.1 | 99 | 5.9 | 4.5 | 0.4 | 0.2 |
